# LEM+ dataset: For agricultural remote sensing applications

**DOI:** 10.1016/j.dib.2020.106553

**Published:** 2020-11-21

**Authors:** Lucas Volochen Oldoni, Ieda Del'Arco Sanches, Michelle Cristina A. Picoli, Renan Moreira Covre, José Guilherme Fronza

**Affiliations:** Earth Observation and Geoinformatics Division, National Institute for Space Research, São José dos Campos, Brazil

**Keywords:** Field reference data, Time series analysis, Remote sensing, Double crop system, Tropical agriculture

## Abstract

Remote sensing allows obtaining information on agriculture regularly with non-invasive measurement approaches. Field data is crucial for adequate agricultural monitoring by remote sensing. However, public available field data are scarce, mainly in tropical regions, where agriculture is highly dynamic. The present publication aims to support the reduction of this gap. The LEM+ dataset provides information monthly about 16 land use classes for 1854 fields from October 2019 to September 2020 (one Brazilian agricultural year) from Luís Eduardo Magalhães (LEM) and other municipalities in the west of Bahia state, Brazil. The reference data were collected in two fieldworks (March 2020 – first crop season, and August 2020 – second crop season). The boundaries of the fields visited in situ were delimited using Sentinel-2 false color compositions (near infrared - red - green) at 10 m spatial resolution. The land use classes were labeled monthly based on information collected in situ (agricultural land use and photographs) and by visual interpretation of Sentinel-2 false color composition (near infrared - shortwave infrared - red) and MODIS/Terra (Normalized Difference Vegetation Index) time series. The dataset can be useful for the development of new pattern recognition methods for agricultural land use mapping and monitoring, comparison of different classification methods, and optical and SAR remote sensing time series analysis. This dataset contributes to complement previous initiatives [1], [2] to make tropical agriculture field reference data publicly available.

## Specifications Table

**Table:** 

Subject	Agricultural science, Earth science
Specific subject area	Remote Sensing, GIS, Land Use and Land Cover
Type of data	Vector (shapefile)
How data were acquired	Geographic coordinates for each field were acquired during two fieldwork campaigns using Locus Map Pro and Sentinel-2 images as a background. Therefore, field boundaries in shapefile (.shp) format, were delimited using Sentinel-2 images at 10 m spatial resolution as the base. A label for each field and each month during one Brazilian agricultural year was determined based on information collected in situ (visual analysis and photo interpretation), false color composition time series of optical images (MSI/Sentinel-2), and the Normalized Difference Vegetation Index (NDVI) time series (MODIS/Terra, MOD13Q1 product, Collection 6). The shapefiles were created and edited using QGIS. The Sentinel-2 images were downloaded from the Copernicus Open Access Hub and Google Earth Engine.
Data format	Raw and analyzed.
Parameters for data collection	Fields located alongside main, secondary, and farm roads.
Description of data collection	The data collection followed three steps: 1) Two fieldworks campaign (one in the first and another in the second crop season) were conducted to collect geographic coordinates and crop type class of each field. 2) Fields’ boundaries were delimited (created a polygon per field) using Sentinel-2 at 10 m of spatial resolution as a background. In this step, the shapefile was created. 3) Each field polygon was labeled with the land use class for each month between October 2019 and September 2020.
Data source location	City/Town/Region: Luís Eduardo Magalhães, Barreiras, São Desidério, and Correntina State: Bahia Country: Brazil Latitude and longitude (and GPS coordinates, if possible) for collected samples/data: Geospatial information is in the dataset.
Data accessibility	Repository name: Mendeley Data Data identification number: http://dx.doi.org/10.17632/vz6d7tw87f.1 Direct URL to data: http://dx.doi.org/10.17632/vz6d7tw87f.1#file-5ac1542b-12ef-4dce-8258–113b5c5d87c9

## Value of the Data

• This dataset, which has monthly land use information over one Brazilian crop year, of 1854 fields, in a tropical agricultural region with a predominant double crop system, is useful as field reference data for remote sensing applications.
• The data can be used for researchers working in agricultural land use classification that need field reference data for training and validating the classification models.
• This dataset can be useful for the improvement or development of new pattern recognition methods for agricultural land use mapping and monitoring; and a benchmark for comparison of different classification methods.

## 1 Data Description

**Fig. 1:**
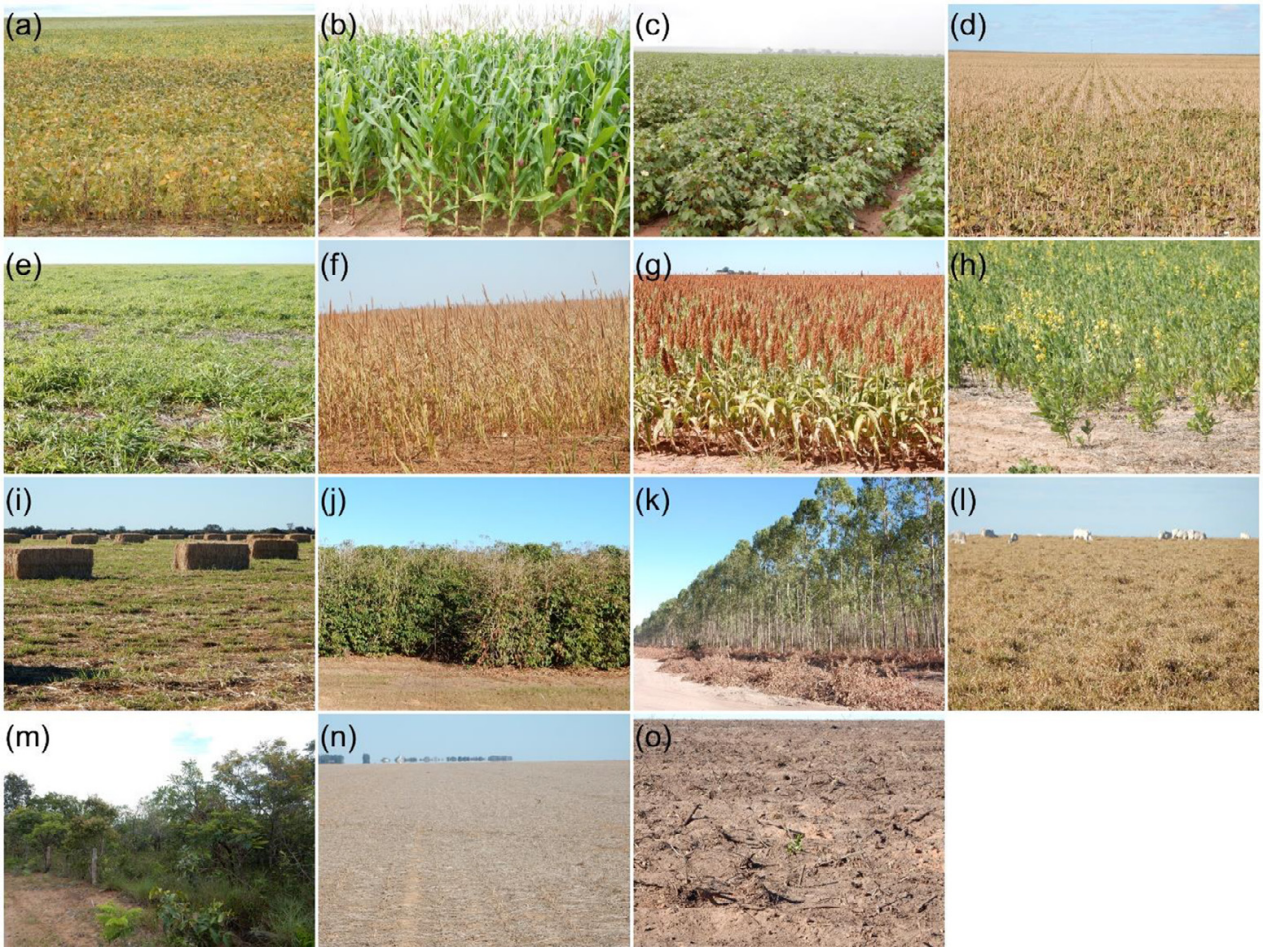
Examples of land use classes of the dataset: (a) soybean, (b) corn, (c) cotton, (d) beans, (e) brachiaria, (f) millet, (g) sorghum, (h) crotalaria, (i) hay, (j) coffee, (k) eucalyptus, (l) pasture, (m) cerrado (natural vegetation of the Cerrado biome), (n) uncultivated soil, and (o) conversion area. There are no images from the "Not identified" class.

**Fig. 2:**
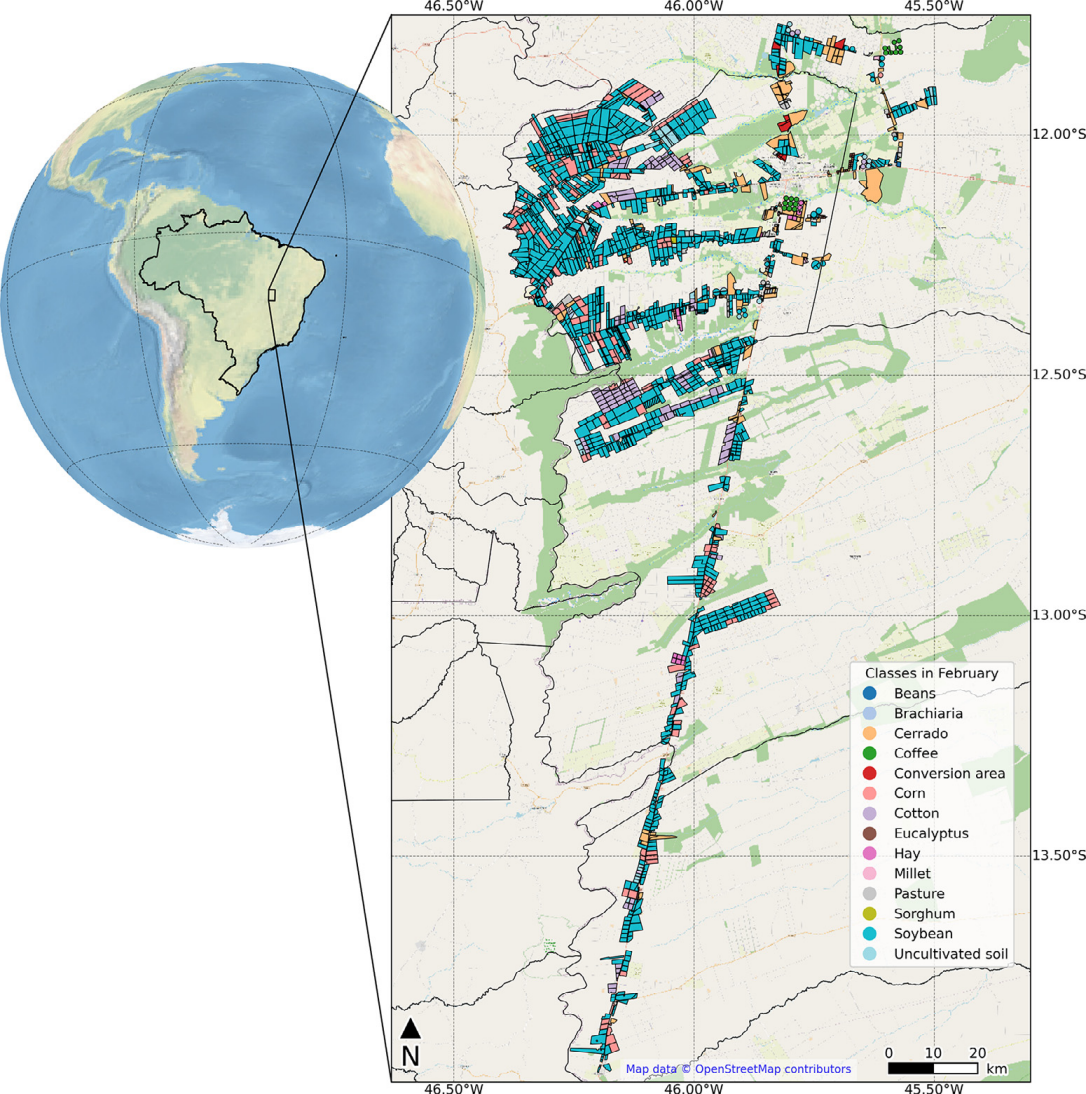
Location of field data collection in west of Bahia state, Brazil, and the distribution of the fields.

**Fig. 3:**
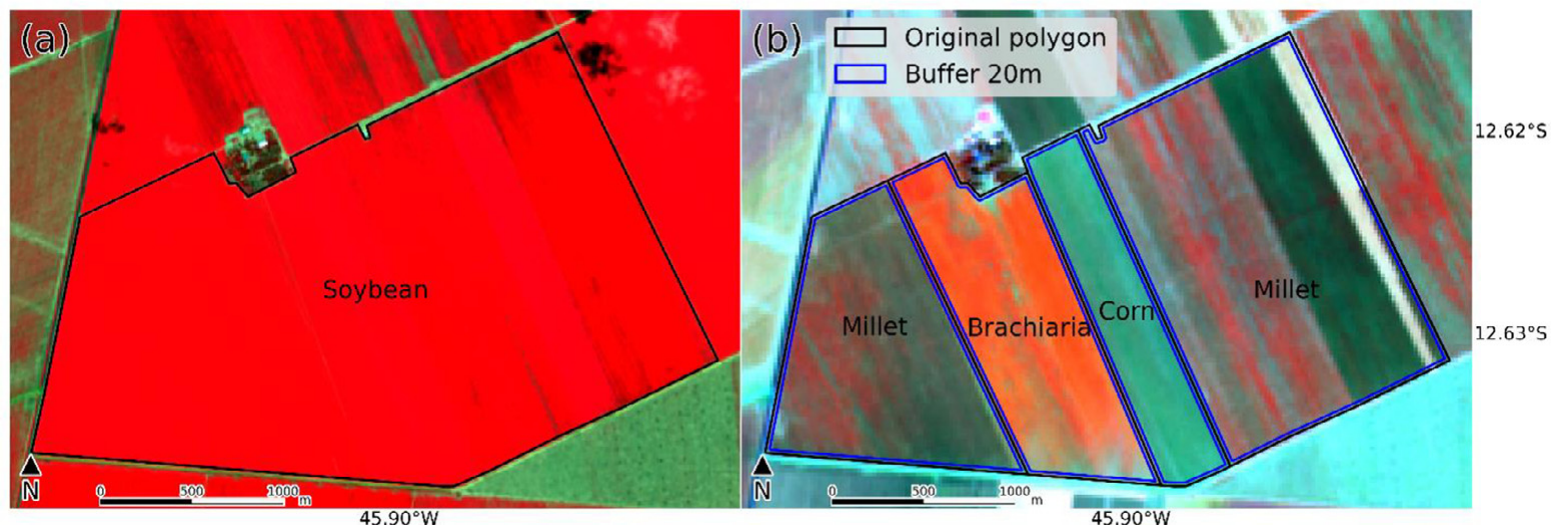
Example of crop field boundaries delimitation over false color compositions of (a) Sentinel-2/MSI (NIR-red-green) of January 30, 2020 at 10 m spatial resolution, and (b) Sentinel-2/MSI (NIR-SWIR-red) of July 13, 2020 at 20 m spatial resolution. In (a) the delimitation from the first fieldwork, and (b) the sub delimitation from the second fieldwork.

**Fig. 4:**
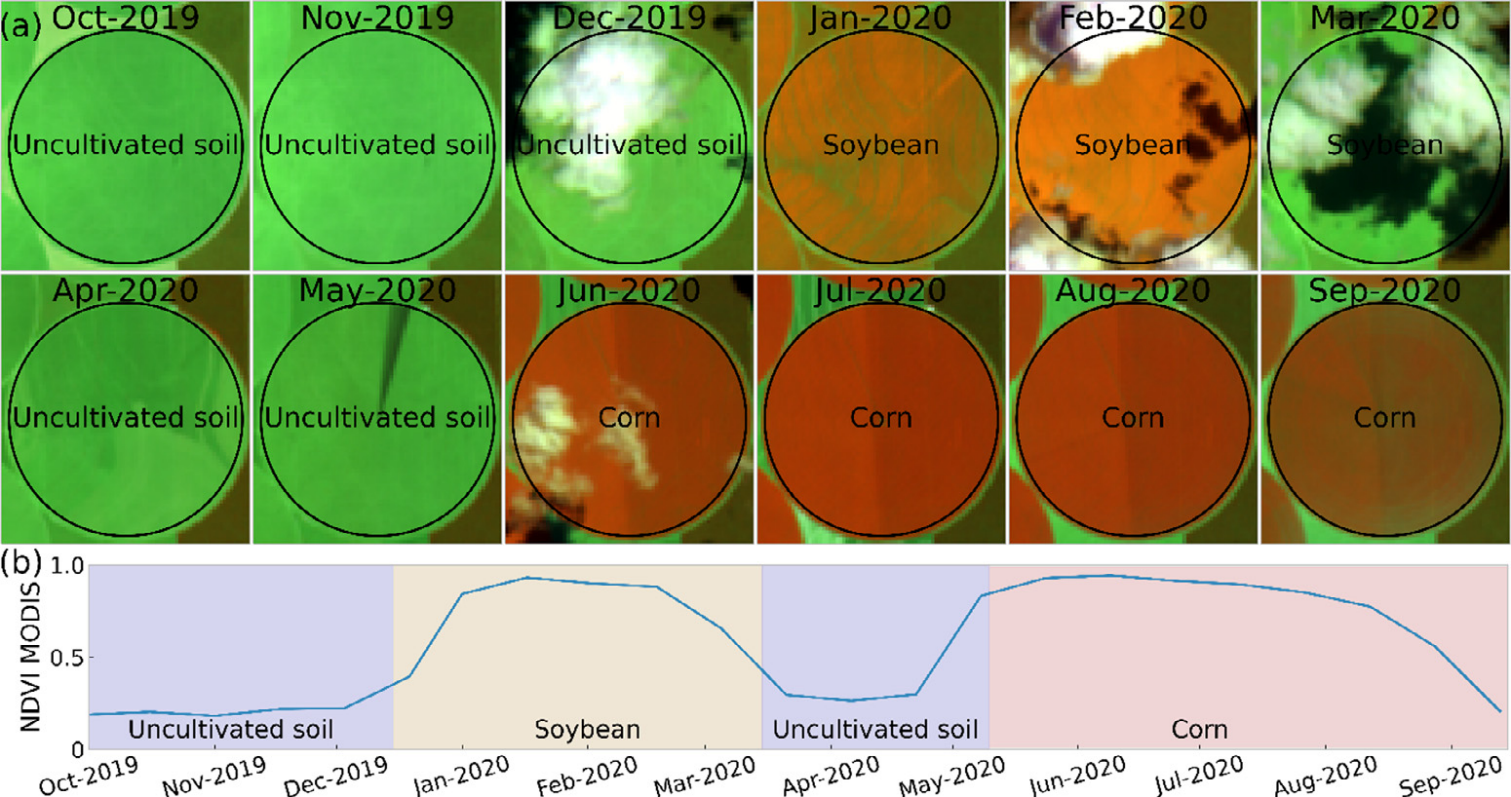
Example of a time series of (a) Sentinel-2 false color composites images and (b) NDVI MODIS used to label the monthly land use class for the field id 1701.

This paper reports the LEM+ dataset, formed by vector file (deposited at http://dx.doi.org/10.17632/vz6d7tw87f.1), in ESRI shapefile format (spatial reference system: WGS 84, EPSG: 4326). The ESRI shapefile format is formed for four files (.shp – the main file that stores the feature geometry, .shx – the index file that stores the index of the feature geometry, .dbf – the dBASE table that stores the attribure information, and .prj – the file that stores the coordinate system information), stores a nontopological geometry and attribute information [3], and can be opened in the majority of GIS software (e.g. QGIS). The shapefile of this dataset includes the delimitation of 1854 fields, and the monthly land use class for each field, from October 2019 to September 2020. There are 14 columns in the attribute table. The first column is the identifier (*id)*, a unique identifier for each field. The next 12 columns are labeled “Month_year” and represent the land use class for that field in that specific month. The last, called "Note", is intended for additional comments. There are 16 land use classes present in the dataset: soybean, corn, cotton, beans, brachiaria (used for seed harvest or soil improvement), millet, sorghum, crotalaria, hay, coffee, eucalyptus, pasture, cerrado (natural vegetation of the Cerrado biome), uncultivated soil (soil with residuals from the previous crop, bare soil, soil tilled, soil with uncultivated plants), conversion area (a recently deforested area that was previously cerrado), not identified (field with some crop of the previous crop year and not yet harvest in October and/or November 2019, and fields with some crop of rapid phenological development between April and June 2020 that could not be identified in the fieldworks). The class names followed a previously published database [1]. Fig. 1 shows photographs take in situ of each land use classes. Fig. 2 shows a map location of data collection with distrubuition of the 1854 fields. Fig. 3 shows examples of field boundaries (fields of id 1837, 1838, 1839, and 1854), over false color compositions of Sentinel-2/MSI (NIR-red-green) (Fig. 3a), and Sentinel-2/MSI (NIR-SWIR-red) (Fig. 3b) as background. Fig. 4 shows the monthly classes for the field 1701, over Sentinel 2/MSI false color composite (NIR-SWIR-red) (Fig. 4a) and NDVI MODIS time series (Fig. 4b).

## 2 Experimental Design, Materials and Methods

### 2.1 Area of data collection

The data were collected in four municipalities in the west of Bahia state, Northeast of Brazil: Luís Eduardo Magalhães (LEM), Barreiras, São Desidério, and Correntina (Fig. 2). These municipalities combined comprise an area of 38,748.4 km². The region presents a tropical wet and dry climate (Aw) according to the Köppen–Geiger classification [4]. These municipalities are inserted in the MATOPIBA region, the newest Brazilian agricultural frontier, which comprises parts of Maranhão (MA), Tocantins (TO), Piauí (PI), and Bahia (BA) states. Usually, one or two harvests are cultivated in one agricultural year in these municipalities. The first crop season occurs in the wet period, from October to March. The second crop season occurs in the dry period, from March to September.

### 2.2 Field data collection

Two fieldwork campaigns were conducted for data collection. LEM municipality was the main area for data collection, but we also took the opportunity to collect information from other points along the way to LEM, and from some fields that were nearby LEM (neighboring municipalities). The first fieldwork, conducted between 2 and 7th March 2020, aimed to acquire information about the first crop season. The second, conducted between 20 and 24th August 2020, aimed to collected information about the second crop season. In each fieldwork, geographic coordinates, crop type, and photographs were collected alongside main, secondary, and farm roads. The information was collected using the Locus Map Pro on a tablet. In each fieldwork, Sentinel-2 false color composition (near infrared (NIR) - shortwave infrared (SWIR) - red) images were used as a background to guide the collection of in situ data (Fig. 3b).

### 2.3 Crop fields boundaries delimitation

After the fieldwork, we delimited the boundaries of the fields visited in situ, using as base mosaics of Sentinel-2 images at 10 m spatial resolution with false color compositions (MSI B8(NIR)-B4(red)-B3(green)) and a linear stretch of 2% (Fig. 3a). Each mosaic was formed with six MGRS tiles (23LLG, 23LMG, 23LLF, 23LMF, 23LLE, and 23LME). Three and one mosaics were used for the first (21 December 2019, 15 January 2020, and 30 January 2020) and the second crop season (28 June 2020), respectively. These images were downloaded from the Copernicus Open Access Hub (https://scihub.copernicus.eu/dhus/#/home).

All fields in the dataset were visited in both fieldworks. Sometimes, a field that had a unique class in the first crop season (Fig. 3a) needed to be subdivided in the second crop season (Fig. 3b). Therefore, the final polygons (the ones available in this dataset) for all the months have the smallest subdivision. To avoid errors in the edges, a 20 m (2 pixels) buffer was applied inside the polygon boundaries (Fig. 3b).

### 2.4 Land use classes

All the fields were labeled with the land use class, monthly, from October 2019 to September 2020, using data collected in the two fieldworks, and visual interpretation of Sentinel-2 false color composition (NIR-SWIR-red) time series at 20 m spatial resolution (Fig. 4a), and MODIS NDVI time series (Fig. 4b). For this step, 12 monthly mosaics of Sentinel-2 were downloaded from the Google Earth Engine. Each mosaic was formed with four MGRS tiles (23LLG, 23LMG, 23LLF, and 23LLE). The first image with less than 20% of clouds was selected for each tile in each month. So, a monthly mosaic could be composed of images of different dates in each tile. The images MODIS NDVI (MOD13Q1 product, Collection 6) were downloaded from www.modis.cnptia.embrapa.br.

## CRediT Author Statement

**Lucas Volochen Oldoni:** Conceptualization, Methodology, Investigation, Visualization, Validation, Writing - Original Draft, Writing - Review & Editing. **Ieda Del'Arco Sanches**: Conceptualization, Methodology, Supervision, Writing - Review & Editing, Funding acquisition. **Michelle Cristina A. Picoli**: Conceptualization, Methodology, Supervision, Writing - Review & Editing. **Renan Moreira Covre:** Investigation, Writing - Review & Editing. **José Guilherme Fronza**: Investigation, Writing - Review & Editing.

## Declaration of Competing Interest

The authors declare that they have no known competing financial interests or personal relationships which have or could be perceived to have influenced the work reported in this article.
